# Feasibility of a web-based program for secondary prevention of suicidal behavior: The iFight Depression-Survive

**DOI:** 10.1192/j.eurpsy.2023.1116

**Published:** 2023-07-19

**Authors:** M. Elices, I. Canosa, A. Toll, A. Justicia, F. Colom, V. Pérez

**Affiliations:** 1 Centro de Investigación Biomédica en Red (CIBERSAM), Madrid; 2 Instituto Mar de Investigaciones Médicas (Imim), Barcelona, Spain

## Abstract

**Introduction:**

Suicide is a leading cause of preventable death in the world. Interventions that can quickly reach a large and geographically dispersed population are needed. Web-based programs are potentially cost-effective, allowing continuity of care. The iFightDepression-SURVIVE (iFD-Survive) is a web-based program designed as an add-on to iFightDepression, a tool developed by the European Alliance Against Depression to target depressive symptoms (https://ifightdepression.com/en/). iFD-Survive is based on dialectical behavioral therapy skills and includes four modules: a safety plan, mindfulness, emotion regulation, and distress tolerance. The content is presented in various formats, including audio, videos, and registers. In addition, weekly telephone support is offered by a mental health nurse.

**Objectives:**

To investigate the feasibility (acceptability, usability, and satisfaction) of the iFD-Survive.

**Methods:**

30 participants who received the intervention as part of a large RCT completed an online survey. To receive the intervention, participants needed to meet the following criteria: 1) digital literacy, 2) having attempted suicide in the last month, and 3) PHQ-9 scores above 5. The online survey included an ad-hoc questionnaire to collect socio-demographic data and data regarding participants’ opinions on the program’s content. The following instruments were also administered:

System Usability Scale (SUS)

Credibility of analogue of therapy rationales

**Results:**

Most respondents were women (20/30), with a mean age of 44 years, and secondary studies (15/30). Most participants (57%) used a mobile phone to access the website and regarded it as “easy to use” (53%). According to the SUS, many of them (57%) reported that they would like to use it frequently and that the tool was “easy” and “safe” to use (53%). Regarding acceptability, 47% of the sample indicated that the iFD-Survive content was adequate to improve their symptoms, and 56% considered that their symptoms of depression have improved as a result of the intervention. 83% of the sample considered telephone follow-up “very useful.” The majority (70%) consulted the program once a week. The “safety plan” and the mindfulness module were regarded as the most useful, followed by “distress tolerance.” The audio for practicing mindfulness skills and the written material were considered very useful, while the videos were valuable.

**Conclusions:**

Online tools can promote continuity of care, helping to prevent further suicide attempts in vulnerable populations. These preliminary findings suggest that the iFD-Survive is feasible among participants with depressive symptoms who have recently attempted suicide. However, these results are based on a small sample of highly educated women; therefore, future research is needed to determine if these can be transferred to other sub-populations.

**Disclosure of Interest:**

None Declared

